# Cardiac regenerative capacity is age- and disease-dependent in childhood heart disease

**DOI:** 10.1371/journal.pone.0200342

**Published:** 2018-07-25

**Authors:** Alexandra Traister, Rachana Patel, Anita Huang, Sarvatit Patel, Julia Plakhotnik, Jae Eun Lee, Maria Gonzalez Medina, Chris Welsh, Prutha Ruparel, Libo Zhang, Mark Friedberg, Jason Maynes, John Coles

**Affiliations:** 1 Labatt Family Heart Centre, Hospital for Sick Children, Toronto, Ontario; 2 Department of Anaesthesia and Pain Medicine, Hospital for Sick Children, Toronto, Ontario; Universita degli Studi di Parma, ITALY

## Abstract

**Objective:**

We sought to define the intrinsic stem cell capacity in pediatric heart lesions, and the effects of diagnosis and of age, in order to inform evidence-based use of potential autologous stem cell sources for regenerative medicine therapy.

**Methods:**

Ventricular explants derived from patients with hypoplastic left heart syndrome (HLHS), tetralogy of Fallot (TF), dilated cardiomyopathy (DCM) and ventricular septal defect (VSD) were analyzed following standard *in vitro* culture conditions, which yielded cardiospheres (C-spheres), indicative of endogenous stem cell capacity. C-sphere counts generated per 5 mm3 tissue explant and the presence of cardiac progenitor cells were correlated to patient age, diagnosis and echocardiographic function.

**Results:**

Cardiac explants from patients less than one year of age with TF and DCM robustly generated c-kit- and/or vimentin-positive cardiac mesenchymal cells (CMCs), populating spontaneously forming C-spheres. Beyond one year of age, there was a marked reduction or absence of cardiac explant-derivable cardiac stem cell content in patients with TF, VSD and DCM. Stem cell content in HLHS and DCM strongly correlated to the echocardiographic function in the corresponding ventricular chamber, with better echocardiographic function correlating to a more robust regenerative cellular content.

**Conclusions:**

We conclude that autologous cardiomyogenic potential in pediatric heart lesions is robust during the first year of life and uniformly declines thereafter. Depletion of stem cell content occurs at an earlier age in HLHS with the onset of ventricular failure in a chamber-specific pattern that correlates directly to ventricular dysfunction. These data suggest that regenerative therapies using autologous cellular sources should be implemented in the neonatal period before the potentially rapid onset of single ventricle failure in HLHS or the evolution of biventricular failure in DCM.

## Introduction

Delineation of the regenerative potential of the heart during the evolution of ventricular dysfunction is key to implementing innovative regenerative therapies more effectively in childhood heart disease. Previous studies have highlighted the inherently more potent regenerative capacity of the neonatal heart [1], leading to early regenerative medicine trials focusing on the congenital heart disease hypoplastic left heart syndrome (HLHS). HLHS is characterized by extreme underdevelopment of the left ventricle (LV), resulting in a mortality rate at one year approaching 25%, despite current surgical treatment [2]. The most common cause of death is failure of the morphological right ventricle (RV), which is recruited as the systemic ventricle in all contemporary surgical options. Phase I and II clinical trials in HLHS found a modest efficacy of autologous regenerative cell therapy in improving RV function [2, 3], suggesting an inherent, but limited endogenous myocardial regenerative capacity. Sano *et al* recently reported the results of 41 single ventricle patients receiving transcoronary infusion of cardiosphere-derived cells (CDCs) during staged surgical palliation [4]. CDC therapy was associated with a reduction in adverse cardiac events relative to untreated, no-placebo controls, but no improvement in all-cause mortality at 2 years follow-up. Subgroup analysis indicated a survival benefit only in patients with reduced ejection fraction (EF) at baseline, whereas there was no difference observed in those with preserved ejection fraction, attributed to more severe fibrotic remodeling in the latter subgroup. Although these data suggest the likelihood of a CDC-mediated regenerative signal, the effects of donor age and morphological diagnosis on the efficacy and durability of autologous CDC infusion remain unknown.

Clinical trials in HLHS have used autologous intra-myocardial or transcoronary delivery of spheres of cells (C-spheres) or C-sphere-derived cells (CDCs), which form spontaneously *in vitro* from atrial tissue explants taken from the same patient at the date of an earlier surgical intervention. C-spheres and CDCs feature high proportions of cells expressing cardiac lineage mesenchymal markers (i.e. vimentin) and transcription factors (GATA4, MEF-2C, Tbx5, and Nkx2.5), and are referred to as cardiac mesenchymal cells (CMCs) [3], but are inherently a mixed population of cells at different stages along the heart developmental pathway. The therapeutic rationale of using the mixed population present in the total unfractionated C-sphere/CDC population results from their potential salutary combinatorial effects and the capacity to generate divergent cell types, including endothelial and vascular smooth muscle cells, fibroblasts and cardiomyocytes.

Mishra *et al* have demonstrated that progenitor cells isolated from the atria of patients with congenital heart disease showed the highest expression of the stem cell markers c-kit and Nkx2.5 in the neonatal age group (compared to infants and older children), and that this cell content directly correlated to their regenerative potential in mouse infarct models [5]. Recent work using kit allele lineage tracing has questioned the capacity of c-kit-positive CPCs to generate *de novo* cardiomyocytes [6], indicating instead that endothelial cells represent the principal fate of kit lineage-traced cells [7]. On the other hand, Ellison *et al* provided evidence based on genetic murine models and cell transplantation experiments that endogenous c-kit-positive cells cardiac stem cells are capable of generating new cardiomyocytes that are necessary and sufficient for myocardial repair [8]. While the debate remains concerning the precise cellular origin of stress-induced cardiomyogenesis, neonatally-derived c-kit-positive cells, and their corresponding secretomes, have been shown to exert potent regenerative effects in preclinical heart failure models [1]. The abundance and regenerative potential of progenitor cells irrespective of their inherent lineage differentiation capacity declines rapidly with increasing age, suggesting that neonatal-derived cells may provide superior therapeutic benefit [1, 9]. Despite the optimism for cell-based therapeutic trials, the baseline regenerative cell content, and any alterations that may occur with the decline in ventricular function, have not been characterized in HLHS, representing a critical knowledge gap to be addressed in the rational design of further therapeutic trials.

To guide further regenerative therapeutic trials in heart failure, we sought to define how the regenerative cellular content changes during cardiac maturation (neonate to child), and with changes in heart function, using ventricular tissue obtained at the time of surgical interventions in HLHS patients, and at the time of transplant in patients with dilated cardiomyopathy (DCM), and during elective surgical repair of TF and VSD characterized by preserved biventricular function. Our data reveal a rapid depletion of stem cell capacity in the RV of HLHS patients, correlating to a decrease in ventricular function. More broadly, our results show for the first time, that progenitor cell content obtained from ventricular explants correlates with ventricular contractile function in both congenital structural and degenerative cardiomyopathies.

## Materials and methods

### Tissue samples

Human tissue explants (~5 mm3) were derived from ventricular biopsies from patients undergoing heart surgery. Approval by the Human Research Ethics Board of the Hospital for Sick Children and written parental consent were obtained for this study (IRB #1000032470). Isolated tissue explants were washed with Iscove’s modified Dulbecco’s medium (IMDM, Gibco, Invitrogen Corporation, Carlsbad, Calif) containing penicillin and streptomycin and supplemented with 10% fetal bovine serum (FBS, Gibco) and then incubated on plastic 12 well plates (Greiner Bio-one) with glass cover slips (VWR) pretreated with 0.1% Gelatin solution (Sigma) for at least 1 hour at 37°C and 5% CO2. Following 2 weeks in tissue culture of explants grown in 10 cm plates the relative cardiosphere (C-sphere) abundance was determined. After a period of 2 to 3 weeks a layer of adherent cells was generated from adherent cardiac spheres (C-sphere-derived cells, or CDCs). The adherent cells were treated with accutase (Innovative cell technologies), washed with IMDM containing penicillin and streptomycin and supplemented with 10% fetal bovine serum, and re-plated on a new 12-well plates with glass cover slips to perform immunohistochemical analysis. C-spheres and C-sphere-derived cells were imaged using a Nikon TE-2000 inverted tissue culture phase contrast microscope. The mounted sections were observed using a Spinning Disk Confocal Microscope and analysed using Volocity software (Volocity software systems Ltd).

### Immunohistochemical analysis

Cultured cells on coverslips or intact myocardial tissues, six-micrometer thick cryosections, were maintained in culture for 2 weeks and then fixed with 4% paraformaldehyde for 20 min at room temperature. Following rinsing with PBS, cells were permeabilized with 0.1% Triton-X 100 (Sigma) for 10 min and then blocked with 5% milk for 30 min and subjected to immunostaining. The following primary antibodies were used in this study: rabbit polyclonal anti-vimentin (Abcam), mouse monoclonal anti myosin heavy chain-β (Clone MF-20, provided by Dr. Donald A. Fischman, Cornell University Medical College, NY), mouse monoclonal anti α-actinin (A7811, Sigma, Inc), rabbit polyclonal anti-ki-67 (Millipore), mouse monoclonal anti-Fsp-1 (Clone 1B10, Novus Biological), mouse monoclonal anti c-kit (PE) (Clone Ab 81, Santa Cruz), rabbit polyclonal anti-fibronectin (H-300, Santa Cruz), rabbit polyclonal anti-MEF-2C (C-21, Santa Cruz), rabbit polyclonal anti-CD31 (Abcam), rabbit polyclonal anti-vWF (Dako Cytomation), and mouse monoclonal anti-desmin (B-7, Santa Cruz). Nuclei were stained with 4,6-diamino-2-phenylindole (DAPI) (D9564, Sigma, Inc).

To measure cardiomycyte proliferation, 0.3micron stacks were Z-projected by summing pixel intensities, then background subtracted in ImageJ. Then each channel was intensity-thresholded in CellProfiler 3.0 to identify cardiomyocyte area (marked by α-actinin), nuclei (DAPI), and phospho-histone (PH3) signal. A constant threshold was applied to the PH3 channel to avoid false positives in fields of view without PH3 signal. Nuclei containing PH3 signal above threshold were considered PH3 positive. Furthermore, PH3+ nuclei with minimum 90% cardiomyocyte overlap were considered PH3+ cardiomyocytes. Counts were normalized to total nuclei per field of view, shown as mean + SEM, and compared by one-way ANOVA and post-hoc Tukey's pairwise comparisons test.

### Echocardiography

RV function was determined using measurement of tricuspid annular plane systolic excursion (TAPSE) and RV fractional area change (RVFAC) using a commercially available ultrasound platform (iE33, Philips Medical Systems, Andover, Massachusetts). All TAPSE data were measured by a single experienced observer blinded to patient clinical status using 3 to 5 consecutive beats and averaged as recommended [1]. These methods have been validated against cardiovascular magnetic resonance (cMRI) as measurements of RV systolic function in children with various types of congenital heart disease[2, 3]. Maximal TAPSE was determined by the total excursion of the tricuspid annulus from its highest position after atrial ascent to the lowest point of descent during ventricular systole in the 4-chamber view, and the measurements converted to z values based on published data acquired from healthy children [10]. The RVFAC value of 36% or above signifies normal RV function [11].

### Statistical analysis

Statistical comparisons relied on a two tailed, type 2 *t*-tests. Data are expressed as mean ± SEM. Results were considered significant at *P* value less than 0.05.

## Results

### Cardiospheres contain pluripotent mesenchymal cells

**Fig 1:**
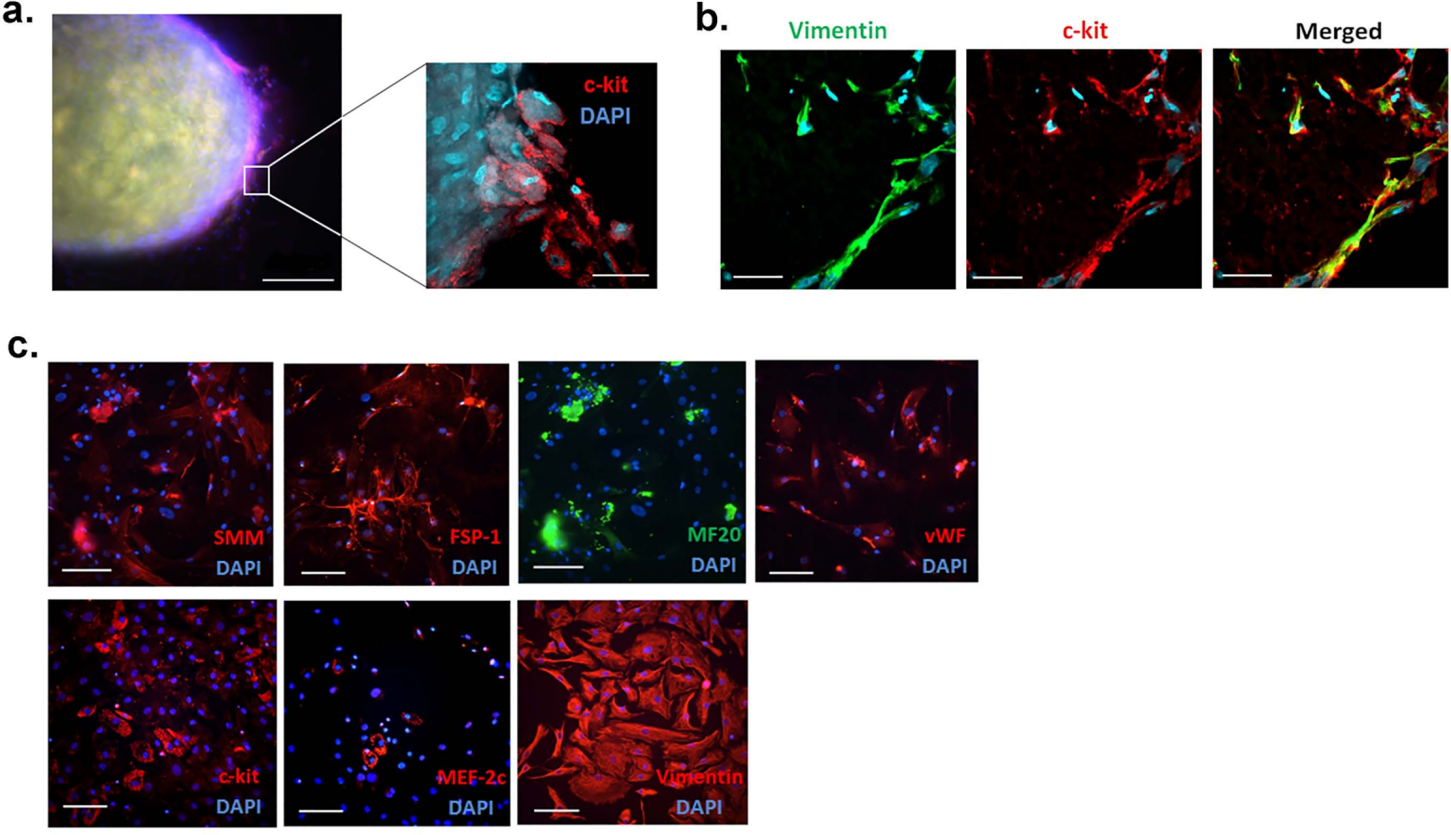
Cardiospheres contain pluripotent mesenchymal cells. **a,** Representative fluorescence microscopy image of intact cardiosphere (C-sphere) spontaneously generated from the RV explant from a patient with tetralogy of Fallot (TF) following 4 weeks in tissue culture. Scale bar, 300 μm. *Inset*, x30 magnified confocal image of c-kit-positive cells (in red) within the shell of the C-sphere. Scale bar, 10 μm. **b**, Representative confocal micrograph of cryosectioned C-sphere obtained from the RV heart sample of patient with TF immunostained for vimentin (in green) merged with c-kit (in red). Scale bar, 20 μm. **c**, Representative images of C-sphere-derived cells immunostained with various differentiation markers, including early cardiogenic marker: MEF-2C, stem cell marker c-kit, the endothelial lineage marker von Willebrands factor (vWF), mesenchymal marker vimentin, fibroblast marker fibroblast-specific protein 1 (Fsp-1), and smooth muscle cell myosin (SMM) marker. Scale bar, 100 μm. Nuclei were marked with DAPI staining (in blue).

**Fig 2:**
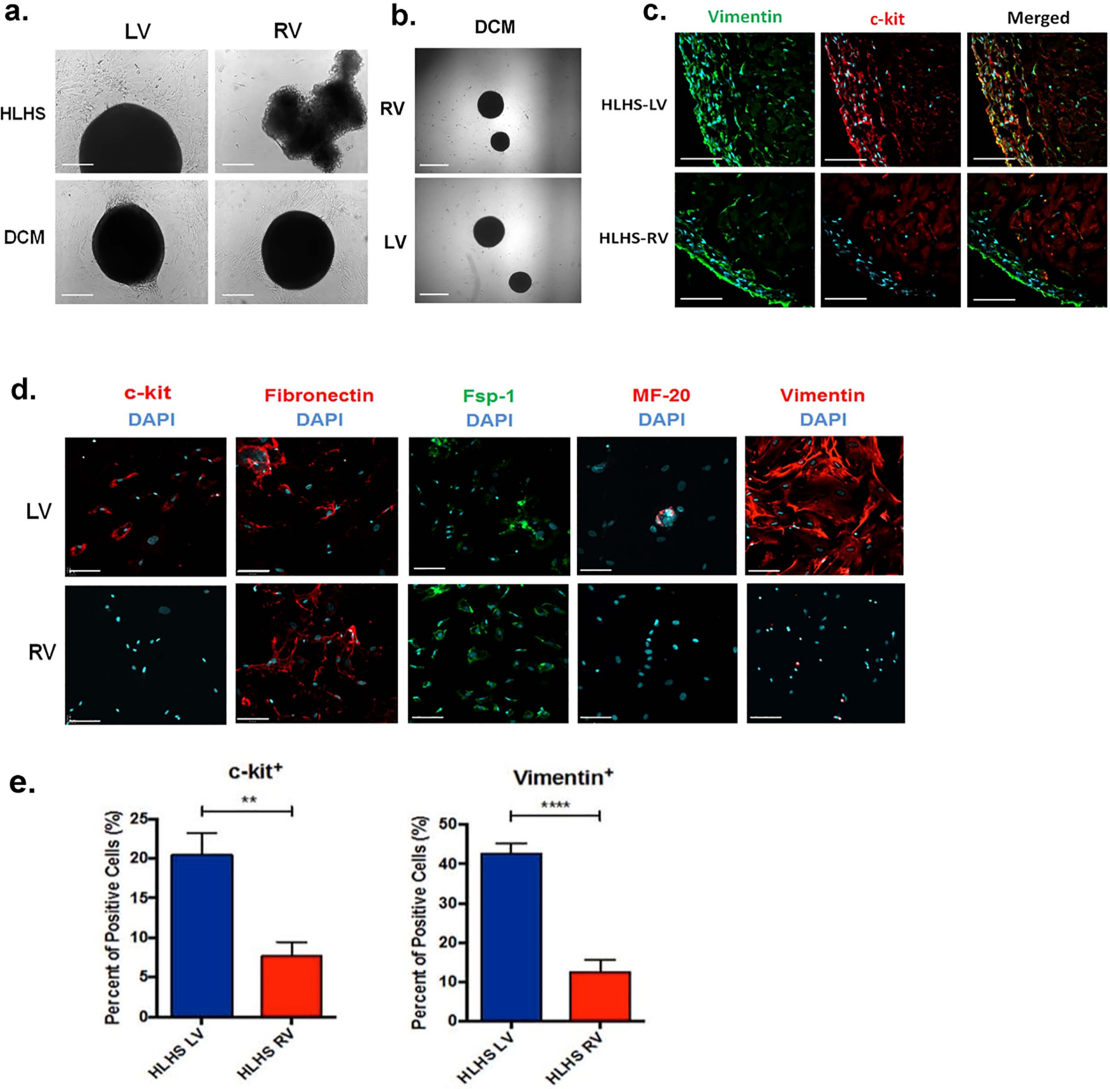
The RV from HLHS exhibits depleted cellular content. **a,** Bright-field images of spontaneously generated C-spheres obtained from HLHS patient (7 month of age) and patient with dilated cardiomyopathy (DCM) (6 months of age). Scale bar, 200 μm. **b,** Bright-field images of C-spheres obtained from both DCM-RV and DCM-LV heart samples propagated in multiple passages. Scale bar, 400 μm. **c**, Representative fluorescence microscopy images of C-spheres derived from the LV and the RV of HLHS patient immunostained for vimentin (in green) and c-kit (in red). Scale bar 100 μm. **d**, Representative fluorescence microscopy images of C-sphere-derived cells (CDCs) obtained from the RV and the LV of end-stage HLHS immunostained for various differentiation markers, including stem cell antigen c-kit, fibronectin, fibroblast marker Fsp-1, early cardiogenic marker (β-myosin heavy chain, MF-20) and mesenchymal cell marker vimentin. Scale bar, 100 μm. Nuclei were marked with DAPI staining (in blue). **e,** Percentage of c-kit and vimentin positive cells in CDCs isolated from HLHS-LV versus CDCs isolated from HLHS-RV. Bar graphs represent mean values ± SEM, n = 10 random fields. c-kit, ** *P* value <0.0018; vimentin, **** *P* value<0.0001.

C-spheres spontaneously developed from myocardial explants within 2–3 weeks under standard culture conditions. As expected, C-spheres did not contain viable functional cardiomyocytes, which were observed to undergo necrosis in the days after tissue removal. The surviving cells that form the C-spheres are more robust to culture conditions, and predominantly feature co-expression of c-kit and the mesenchymal marker vimentin (herein referred to as CMCs) (Fig 1A and 1B), as previously shown in the analysis of C-spheres derived from the atria of patients with congenital heart disease [5] and from adult myocardium [12]. Cardiac mesenchymal cells (CMCs) are the most abundant cardiac stem cell type generated from human myocardial biopsies [12]. CMCs expressing the receptor tyrosine kinase c-kit have been shown to exert potent regenerative capacity in preclinical heart failure models [13, 14]. Since c-kit is a pluripotency marker, the CDCs that emanate from the C-sphere were checked for cell type lineage markers. Mixed populations of CDCs contained cells that expressed markers of endothelial (vWF), fibroblastic (Fsp-1), and smooth muscle (SMM) lineages, and mesenchymal cells that expressed vimentin and c-kit (Fig 1C). We note that populations of CDCs also contained cells expressing the early cardiogenic markers myocyte-enhancer factor-2c (MEF-2C) and sarcomeric β-myosin heavy chain (MF-20) (Fig 2D).

### The regenerative compartment alters with age and ventricular function in HLHS and DCM

In HLHS, the morphologic RV is surgically configured to become the systemic ventricle, while the underdeveloped LV largely remains unloaded and provides minimal or no stroke volume. We found that in patients with end-stage HLHS, the failing RV failed to produce C-spheres in culture (Table 1), despite a young patient age (7 months). In contrast, the RV and LV of young patients with failing DCM (6 months of age) produced a large number of C-spheres, whereas a more aged failing DCM patient (14 years) failed to produce C-spheres (all samples collected at the time of transplantation). By comparison, all RV samples from infant (< 1 year) TF patients generated abundant cellular content in culture, representing all cardiovascular lineages, whereas beyond one year of age derivable stem cell content was very limited or absent (Table 1). These results illustrate an age-dependent depletion of the regenerative compartment evident after one year of age irrespective of disease and ventricular function. Further, the regenerative content and capacity of myocardial explants to generate C-spheres becomes substantially depleted at less than one year of age in HLHS with the onset of ventricular dysfunction.

**Table 1:** Ventricular C-sphere content.

Diagnosis	Age	C-spheres*	Ventricular function
TF	8 months	10	N
TF	3 months	10	N
TF	6 months	5	N
TF	7 months	10	N
TF	9 months	8	N
TF	3.50 years	3	N
VSD	7.0 years	0	N
Coronary artery thrombosis	14 days	10	LVAD
HLHS, Unbalanced AVSD	8 days	4	Died P/O Sano-Norwood, pulmonary hypertension
HLHS	7 months	LV: 10 RV: 2	Transplant RV dysfunction
VSD	6.10 years	RA: 2	N
DCM	6 months	LV: 10 RV: 10	Transplant LV dysfunction
VSD	12.50 years	RV: 1	N
DCM	13.75 years	LV: 0 RV: 0	Transplant Biventricular dysfunction

TF, Tetralogy of Fallot; VSD, Ventricular Septal Defect; AVSD, Atrioventricular septal defect; HLHS, Hypoplastic left heart syndrome; DCM, Dilated cardiomyopathy; N, N = normal; RA, right atrium.

* Relative number of C-spheres per image field in RV unless otherwise noted as LV or RA, representing at least 6 fields per patient sample.

### The right ventricle from HLHS exhibits depleted progenitor cellular content

To address the innate regenerative capacity in HLHS, we isolated C-spheres generated from primary cardiac tissue samples obtained during Stage I surgical repair or at the time of transplantation. We observed a remarkable difference in the cellular phenotypes between C-spheres generated from the failing RV and the contractile but non-ejecting LV at transplantation. The diminutive LV yielded abundant C-spheres, whereas C-spheres derived from the RV were smaller, irregular in shape, and contained fewer viable cells (Fig 2A). By comparison, transplantation specimens from both ventricles in patients with advanced DCM generated abundant C-spheres, similar to that in the LV in HLHS (Fig 2A), and showed multiple passage capability (Fig 2B). In HLHS, an important distinguishing feature of C-spheres generated from the RV, compared to the LV, was the loss of pluripotent c-kit-positive cells at the edges of the spheres (Fig 2C), whereas both LV and RV produced spheres containing vimentin positive cells.

Approximately half of the CDCs generated from LV C-spheres in HLHS expressed the stem cell marker c-kit; the remaining non-c-kit cells expressed the early cardiomyocyte marker MF-20, the mesenchymal marker vimentin, or less frequently, fibroblast markers fibronectin (Fn) or Fsp-1 (Fig 2D). Almost all CDCs generated from the LV expressed the cardiac mesenchymal marker vimentin, whereas vimentin-expressing cells were not found in CDCs obtained from the RV (unlike the C-sphere itself, which did express vimentin) (Fig 2E). CDCs from the RV mainly expressed the fibroblast markers Fsp-1 and Fn (Fig 2D), with a significant reduction in the frequency of c-kit-positive cells (Fig 2E). These results indicate that the contractile but diminutive LV in HLHS maintains the capacity to robustly generate significant C-spheres and CDCs with multilineage potential, whereas the failing RV produces a high proportion of cells committed to a fibroblast phenotype.

**Fig 3:**
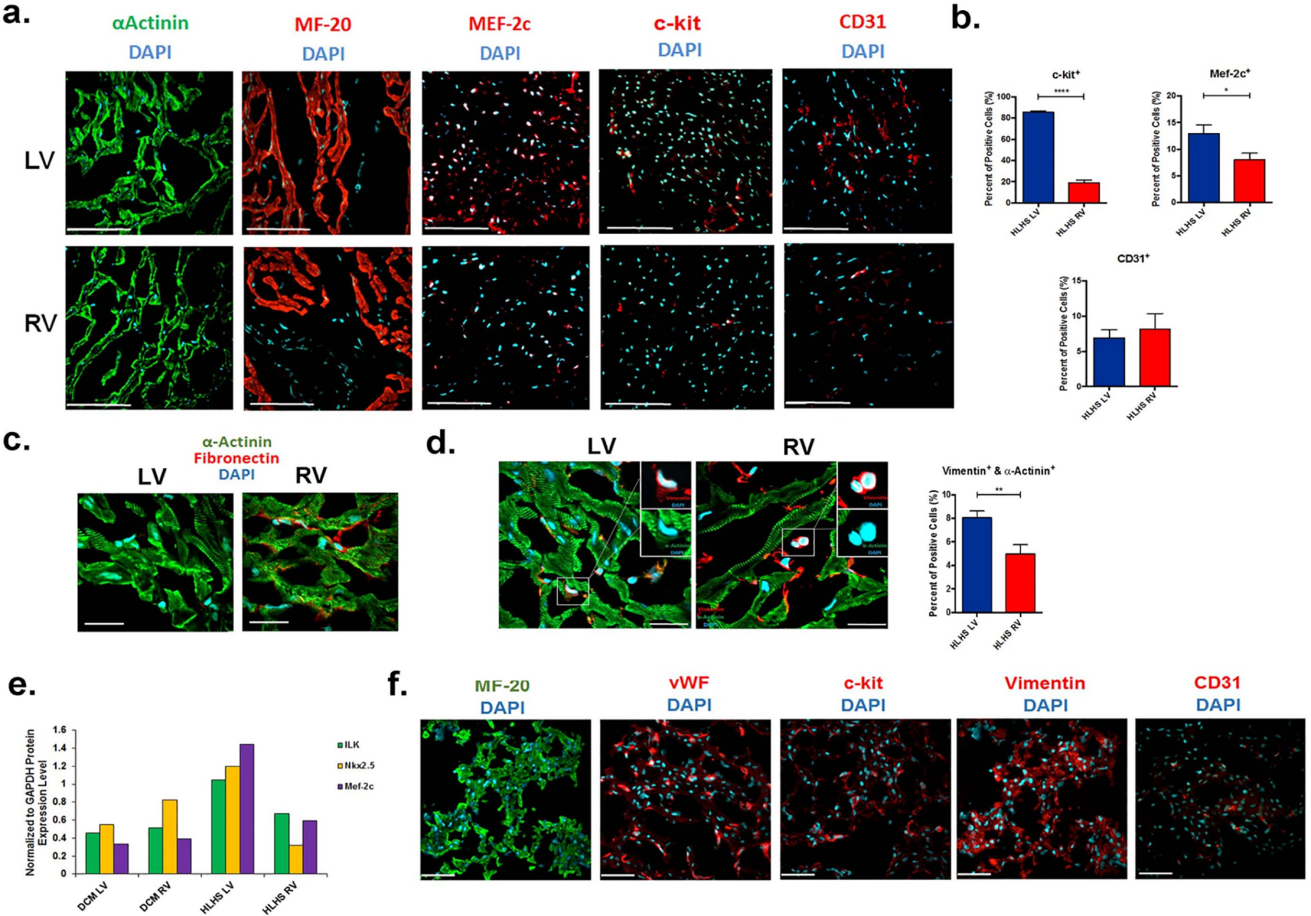
The RV in HLHS becomes deficient in cardiovascular lineage progenitor cells during the evolution of heart failure. **a,** Representative fluorescence microscopy images of cryosectioned HLHS samples showing the cellular content in the RV and the LV at transplantation, immunostained for cardiac markers α-actinin and β-myosin heavy chain (MF20), progenitor cell marker c-kit, the early cardiogenic marker MEF-2C and the endothelial cell marker CD31. Scale bar, 150 μm. **b**, Percentage of c-kit-, MEF-2C- and CD31-positive cells in cryopreserved tissue samples isolated from HLHS-LV versus the HLHS-RV. Bar graphs represent mean values ± SEM, n = 10 random fields. c-kit * *P* value<0.0001; MEF-2C * *P* value<0.029; CD31 *P* value = NS. **c**, Representative confocal micrographs of cryosectioned tissues obtained from the RV and the LV of HLHS patient immunostained for fibronectin (in red) and α-actinin (in green). **d**, Representative confocal micrographs of cryosectioned tissues obtained from the RV and the LV of HLHS patient immunostained for vimentin (in red) and α-actinin (in green). Image insets represent 3 X magnification. Scale bar, 20 μm. Bar graph, percentage of α-actinin- and vimentin-double positive cells in cryopreserved tissue samples isolated from HLHS-LV versus HLHS-RV. Data are mean values ± SEM, n = 10 random fields. ** *P* value<0.0057. **e**, Western Blot analysis of protein expression levels of cardiac lineage markers Nkx.2.5 and MEF-2C, and that of integrin-linked kinase (ILK) in the LV compared to the RV in HLHS, and in RV and LV in DCM (age 6 months). **f**, Representative fluorescence microscopy images of cryosectioned RV cardiac tissue isolated from neonatal patient (age, day 8) immunostained with MF-20, vWF, c-kit, vimentin and CD31 markers. The neonatal RV in HLHS resembled the LV in HLHS at transplantation, based on the abundant presence of c-kit, vimentin, and the endothelial markers (vWF; CD31). Scale bar, 80 μm. Nuclei were labeled with DAPI staining (in blue).

To determine if the cellular phenotypes were a result of culture conditions *per se*, cryopreserved RV and LV myocardium from HLHS was obtained at the time of heart transplantation and analyzed in parallel to culture-isolated cells. Immunostaining of RV and LV cryosections showed similar myocardial structure, revealed by α-actinin and MF-20 staining features. Consistent with the changes observed in the CDC cellular phenotypes, there were significant reductions in c-kit and the early cardiogenic marker MEF-2C expression in the failing RV (Fig 3A and 3B). The LV cyrosections contained significantly more cells that were double-positive for vimentin and α-actinin (Fig 3D), as well as for MEF-2C and cardiomyocyte desmin (not shown), indicating an increase in the number of immature or cells with a nascent cardiac myocyte phenotype within the LV of HLHS, as compared to the RV. Western Blot analysis confirmed higher protein expression levels of cardiac lineage markers (Nkx.2.5 [15] and MEF-2C), as well as that of integrin-linked kinase (ILK) in the LV compared to the RV, which collectively represent both markers and mediators of cardiomyogenesis [16, 17] (Fig 3E). The RV showed increased fibronectin staining (Fig 3C), paralleling the finding that more CDCs with a fibroblast phenotype were obtained from the RV. We did not see a difference in the number of CD31 positive cells between the LV and the RV. These results indicate that the depletion of early cardiac mesenchymal lineage cells and the regenerative compartment occurs in the systemic morphologically RV during the process of heart failure.

To determine if the RV in HLHS is congenitally deficient in regenerative capacity, the cellular constituents derived from the “Sano core” (the RV muscle excised to construct the RV to pulmonary artery conduit), obtained from a newborn HLHS patient at Stage I surgical repair (day of life eight), were analyzed using the same cellular markers (Fig 3F). The neonatal RV was shown to contain populations of c-kit- and vimentin-positive cells (CMCs), as well as endothelial cells, similar to that seen in the LV from the 7 month old HLHS obtained at the time of transplantation. Together, these findings suggest that the depletion of cardiovascular lineage cells observed in the failing RV in HLHS results from the process of RV failure, rather than from an intrinsic lack of cells with regenerative potential within the RV at birth.

### Progenitor cell content correlates with ventricular function

Having determined that the loss of the regenerative cell compartment correlates with ventricular failure of the RV in HLHS (Table 1), we sought to determine if cardiac functional metrics (echocardiography) could more generally be used to determine the quantity/presence of cardiovascular lineage cells in cardiac tissue. The 7-month of age HLHS patient with severe RV dysfunction (TAPSE z-score = -4.3) requiring transplantation exhibited a profound depletion of progenitor cells and minimal production of C-spheres/CDCs, whereas the RV from a neonate (day 8 of life) HLHS patient exhibited preserved ventricular function and abundant cardiac progenitor cells (Table 2). Even with RV failure, the adjacent small but well-functioning LV (preserved LV function, LVFAC = 52%) produced numerous C-spheres and CDCs (Supplementary Movie). A similar aged (6 month) DCM patient requiring transplantation with preserved RV function (TAPSE z-score = 0.09) generated abundant progenitor cells from the RV and the LV. In contrast, RV and LV samples from a 14 year-old DCM patient with severe biventricular failure (TAPSE z-score = -5.1) did not generate C-spheres/CDCs in culture (Table 2). A similar correlation was observed in all patients using RVFAC, which measures RV circumferential strain as an orthogonal measure of global RV function. Qualitative assessments of RV function correlated closely to TAPSE and RVFAC scores (Table 2). These results demonstrate that echo-determined RV and LV function correlate to the stem cell content in the corresponding ventricle, irrespective of age or diagnosis.

**Table 2:** Correlation between echocardiographic ventricular function and stem cell content.

Patients	RV function		LV FAC (%)	Qualitative RV/LV function	Stem cell content	
	TAPSE (z-score)	RVFAC (%)			RV	LV
HLHS (Age: day 8)	z = 1.63	47	N/A (tiny LV)	Good RV function Small contractile LV	Abundant	Absent
HLHS (Age: 7 mo.)	z = -4.3	32	52	Moderate ↓ RV function Small contractile LV	Absent	Abundant
DCM (Age: 6 mo.)	z = -0.9	26	22	Moderate ↓ RV function Severe ↓ LV function	Abundant	Abundant
DCM (Age: 14 yrs.)	z = -5.1	19	3	Moderate-severe ↓ RV function Severe ↓ LV function	Absent	Absent

TAPSE—Tricupsid Annular Plane Systolic Excursion, RVFAC—Right Ventricular Fractional Area Change (%), LVFAC—Left Ventricular Fractional Area Change (%)

### Cardiomyocyte proliferation is not evident in HLHS or TF

**Fig 4:**
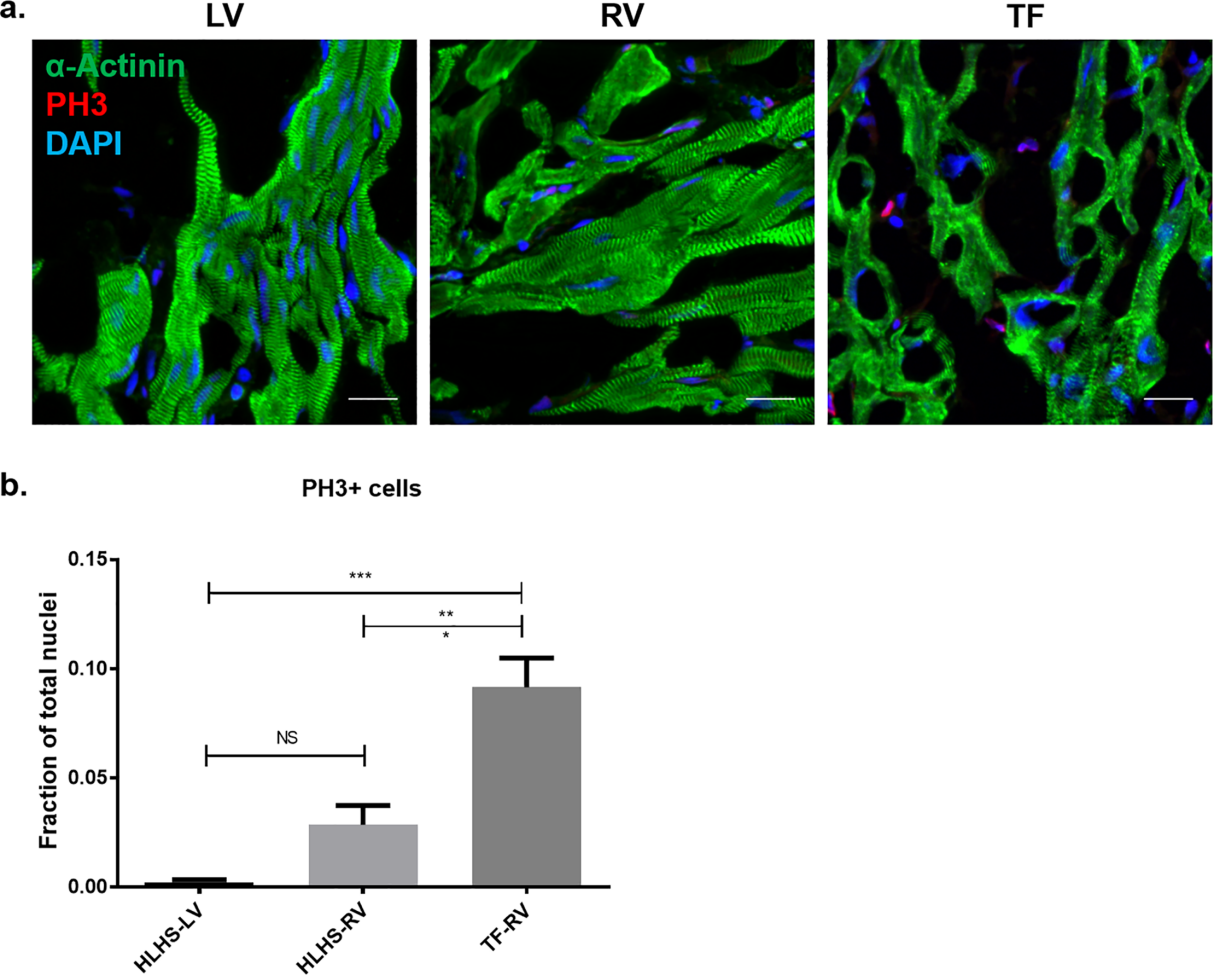
DNA replication occurs in stromal cells but not in existing cardiomyocytes in HLHS and in TF. **a,** ventricular samples from HLHS RV, HLHS LV, and TF immunostained for cardiomyocyte α-actinin and PH3. **b**, the % of non-cardiomyocytes that were PH3-positive was higher in TF than in HLHS. PH3+ nuclei with minimum 90% cardiomyocyte overlap, considered as PH3+ cardiomyocytes (CM), were not detectable in any heart samples. Counts in non-CMs were normalized to total nuclei per field of view, shown as mean + SEM, and compared by one-way ANOVA and post-hoc Tukey's pairwise comparisons test. Scale bar = 20 microns. Blue = DAPI, red = PH3, green = α-actinin.

Cardiomyocyte proliferation block specific to the LV has been implicated in an elegant genetic mouse model of HLHS [18]. Using a large-scale mutagenesis screen, Hisato *et al* identified mutations in *Sap130* and *Pcdha9* which in mouse closely phenocopy the small LV seen in patients with HLHS. The authors implicated a block in cell cycle progression in developing LV cardiomyocytes, evident as increase in phospho-histone 3 (PH3) positive cardiomyocytes in concert with decreased Ki67 immunostaining, which was discernible in the LV but not RV during embryonic mouse development. To evaluate the potential for cardiomyocyte proliferation in human postnatal HLHS myocardium, we quantified the co-expression of cardiac α-actinin and PH3 using IHC in human HLHS samples (Fig 4). This analysis showed absence of DNA synthesis among cardiomyocytes in the LV and RV in HLHS, or within the RV in TF. There was, however, evidence of non-cardiomyocyte proliferation detectable in all heart samples that was significantly more robust in the TF sample compared to that in HLHS.

## Discussion

This study confirms previous findings that myocardial explants from pediatric patients with congenital heart disease and neonatal DCM spontaneously generate C-spheres under standard culture conditions, and that C-spheres can give rise to more differentiated cells representative of the cardiovascular lineage [3, 4, 9]. This result is in keeping with the observation that C-spheres are comprised of cells exhibiting a primitive, pluripotent mesenchymal phenotype. Here, we confirm these general findings in HLHS myocardium, but also highlight dynamic changes in the progenitor cell capacity in the RV in HLHS during the evolution of contractile failure. Specifically, we show for the first time a clear relationship between ventricular systolic function and stem cell content in HLHS and DCM that may have broad significance to regenerative medicine therapy.

The diminutive but non-failing LV in HLHS is shown here to be an unsuspected repository of cells with regenerative capacity, as well as of immature cardiomyocytes, even after the onset of advanced RV failure. In contrast, we note that both the RV and LV in neonatal DCM exhibit abundant C-spheres and CDCs comparable to that in the LV, but not the failing RV, in HLHS. This suggests that RV-centric failure in HLHS results in profound and rapid depletion of regenerative capacity, possibly resulting from a volume- and pressure-loaded morphological RV. This finding is in agreement with the known clinical predisposition of the RV to develop premature failure when configured to deliver blood systemically [19]. In contrast, we propose that the contractile but stroke work-unloaded LV in HLHS retains robust regenerative capacity comparable to other ventricular phenotypes less than one year of age since it is morphologically designed as the systemic ventricle. It is noteworthy that the neonatal RV in tetralogy of Fallot is also subject to elevated pressures, yet invariably demonstrated robust CMC content in culture in the first year of life and the retention of normal RV contractile function.

**Fig 5:**
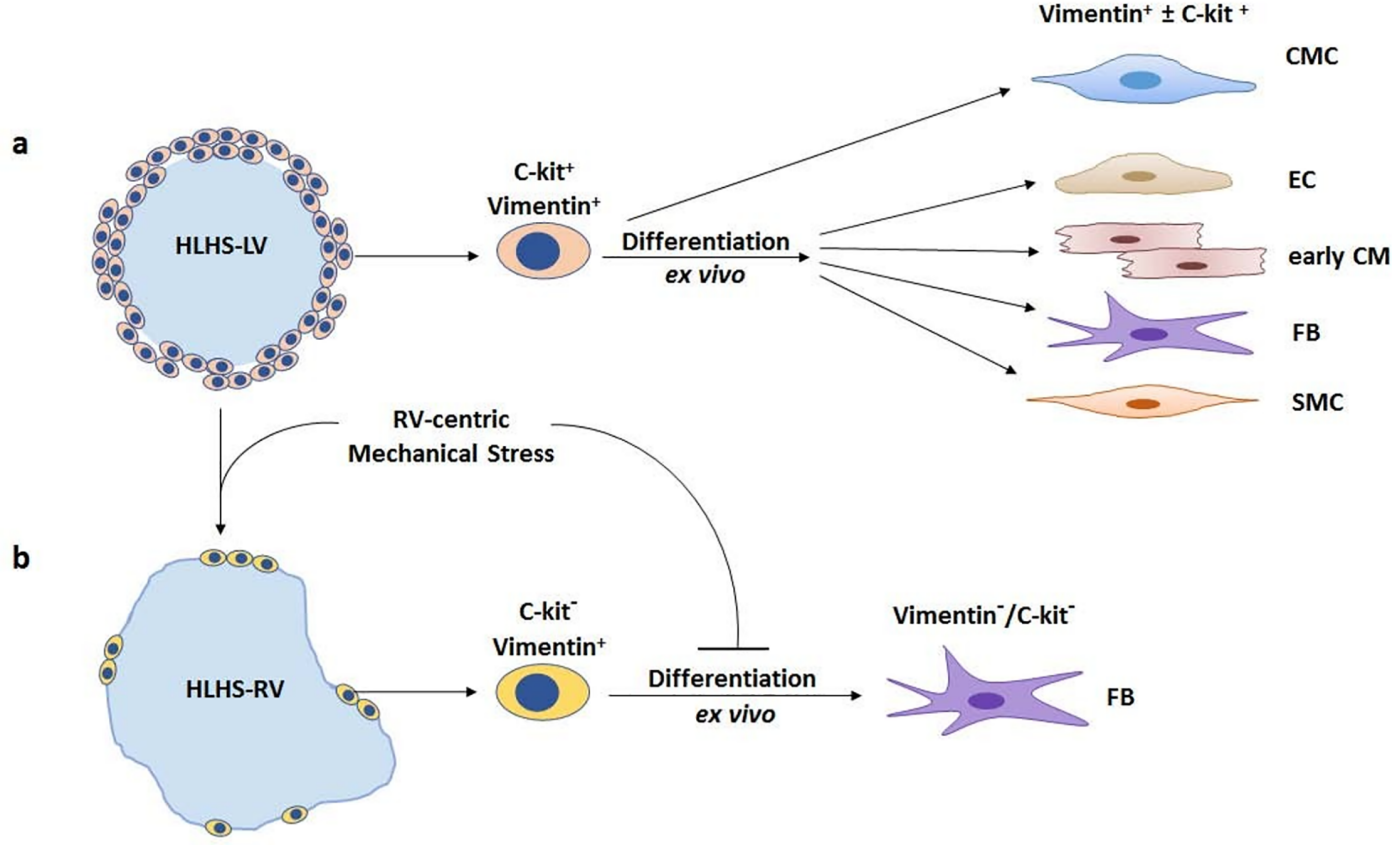
Schematic representation of differentiation capacity in LV and RV in HLHS. **a,** C-spheres generated from LV in HLHS are comprised of continuously layered double-positive c-kit+ and vimentin+ cells. C-spheres generate cardiosphere-derived cells which possess adherence properties consistent with a cardiac mesenchymal cell (CMC), and exhibit differentiation capacity into multiple cardiovascular lineage cell types as shown. Immature cardiomyocytes are more frequent in the LV than the RV. **b,** Excessive RV mechanical stress results in depletion of regenerative capacity in the RV in HLHS. CMCs within HLHS-RV tissue are sparsely distributed on the surface of the tissue fragments and create a non-spherical shape. These cells are vimentin-positive and c-kit^-^negative and become incompetent for differentiation as a result of stress-induced senescence. CMCs, cardiac mesenchymal cells; EC, endothelial cells; CM, cardiomyocyte; FB, fibroblast; SMC, smooth muscle cell.

As our report is the first describing the loss of the regenerative compartment in the process of heart failure, the molecular or physiological mechanisms that lead to a significant reduction in CMCs in the failing ventricle are unknown. Inherent in most forms of heart failure is an imbalance of blood/nutrient demand in relation to delivery, and the coronary circulation after the first stage repair in HLHS (post-Norwood procedure) is vulnerable as a result of elevated ventricular impedance and impaired subendocardial blood flow [20]. Related to these metabolic changes, redox or ischemic stress and a pro-inflammatory microenvironment have been shown to deplete stem cell capacity and impair cardiomyogenic differentiation in mouse models of heart failure [21] and in human samples [22, 23]. Senescence induction due to local inflammation may also underlie the well-recognized failure of survival of transplanted stem cells, and p53 induction can mediate accrued replicative senescence during heart failure [24]. Preservation of cardiac regenerative potential depends upon the retention and viability of the endogenous CMC compartment, which in turn will determine the efficacy of autologous regenerative therapies (Fig 5).

A genetic model of HLHS implicated a block in cardiomyocyte proliferation and differentiation in early (E.14) mouse development as the cause of LV hypoplasia [25]. The potential for cardiomyocyte cell cycle re-entry as a source of new cardiomyocytes has been established in human postnatal myocardium as assessed using radiocarbon methodology [26, 27], although the rate of cardiomyocyte renewal, exclusive of multi-nucleation, is less than 1% per year. Our findings showing a lack of cardiomyocyte PH3 staining in both the RV and LV in HLHS, and in the RV in TF, but easily detectable evidence of proliferation in stromal cells, confirms the findings in previous studies indicating that mesenchymal and endothelial cells, but not cardiomyocytes, are highly proliferative throughout life [27]. Since cardiomyocyte proliferation is normally rare in the human postnatal heart, it remains an unanswered question as to whether human HLHS is directly caused by a cardiomyocyte proliferation block during prenatal LV development.

Regardless of causation, the RV in HLHS showed depletion of CMC content that was not observed to the same degree in the age-matched RV in advanced DCM or in TF, or even in the LV in HLHS obtained contemporaneously from the same patient. The marked depletion in progenitor cell content in the RV suggests that cellular transplantation in HLHS may be more effective than alternative strategies that rely upon activation of endogenous RV regenerative potential, or alternatively, that therapies need to be administered very early in the disease process. However, the finding of abundant C-spheres with multiple passage clonogenicity in advanced DCM suggests that activation of the endogenous regenerative compartment is an attainable rescue approach in DCM at least in the first year of life. It should be noted that hMSCs generate exosomal microRNA cargo [28] and cardiac fibroblasts [29] release soluble factors containing cardioactive factors which may account for overall loss of regenerative capacity resulting from depletion of the MSC compartment observed in this study.

It cannot be determined from our data whether stem cell depletion initiates contractile dysfunction in HLHS and DCM, or whether it simply occurs coincident with the disease process. There is compelling evidence that chemotherapeutic drugs, such as doxorubicin, cause cardiomyopathy due to a direct toxic effect on the stem cell compartment [30]. Our data suggest that stem cell depletion may exacerbate, if not cause, ventricular dysfunction that occurs in both structural and degenerative forms of childhood cardiomyopathy. Regardless of whether stem cell depletion represents the cause or the effect of ventricular remodeling, our data support the rationale of using regenerative therapy to activate or re-populate the stem cell compartment as a strategy to rescue ventricular remodeling in HLHS and in DCM, but that the timing and location of administration of therapies may need to be personalized to achieve the best therapeutic effect.

## Study limitations

Exomic sequencing of the first genetic model of HLHS highlighted the complex multigenic causation, revealing mutations in genes, such as *Sin3A/Sap130* and *Rbfox2*, which are known to affect cardiac progenitor cell survival and differentiation [25]. Deep sequencing of patients in this study was not performed to detect the status of candidate genes such as Notch pathway genes implicated in the genesis of HLHS [25]. However, the results of our study suggest that genetic susceptibility to cardiac stem cell dysfunction may conspire with hemodynamic stress to cause stem cell depletion as observed in human HLHS in the current study.

Second, our conclusions are necessarily based on limited sample sizes. For example, the conclusion that ventricular dysfunction is correlated to myocardial explant stem cell content is based on the analysis of only 5 patients and 8 samples: 2 patients with HLHS (2 with RV, 1 with LV samples), 2 patients with DCM (2 samples each for RV and LV), and one patient with ischemic cardiomyopathy (1 LV sample). The conclusion that marked stem cell depletion occurs after one year of age in the setting of preserved biventricular function is based on 10 patients including 6 with TF (7 < 12 months, 4 > 12 months), 3 patients with VSD (all > 12 months), and a single 14 year-old patient with DCM. However, the overall discriminatory value of one year of age as the threshold for stem cell depletion is high. The error bars and statistical data shown in Figs 2 and 3 are based on comparison of the RV to the LV in a single patient and represent number of fields and not number of patients counted.

Third, we note that the HLHS patient with RV-centric failure requiring transplantation exhibited a small but contractile LV, whereas many patients with HLHS, characterized by combined aortic and mitral atresia, present a more completely underdeveloped LV.

Fourth, we assumed that C-sphere abundance was a global surrogate for endogenous stem cell capacity, whereas there is much unresolved complexity regarding the nomenclature, phenotypic identity, and regenerative potential of the various constituent stem cells present in the human heart. The regenerative capacity of isolated stem cells was not tested using *in vivo* mouse MI cell transplantation models, however, the remodeling process following MI is likely to differ from that observed in HLHS and neonatal DCM. Therefore, more detailed sub-analyses of putative stem cells acquired in larger numbers from age- and disease-diverse patients will be required for more complete understanding of the endogenous stem cell compartment in pediatric heart disease, as will evaluation of the *in vivo* therapeutic potential of autologous cellular transplantation.

## Conclusions

Analysis of pediatric heart disease samples in this study provides the first evidence that depletion of the endogenous stem cell capacity occurs during ventricular failure, and correlates with echocardiographic functional parameters for both the LV and the RV. We demonstrate that explants from congenital heart disease myocardium are, in general, a lucrative *ex vivo* source of cardiovascular lineage cells in the first year of life. Our findings support the therapeutic premise of pre-emptive stem cell therapies using autologous cells that are obtained prior to the development of advanced ventricular failure. Further, the abundant progenitor cell content derivable from both ventricles even in advanced DCM suggests that activation of the endogenous stem cell compartment is a viable strategy in neonatal DCM. Beyond the neonatal period, any benefit resulting from regenerative therapies is likely to result from paracrine effects directed to existing myocardium and not from activation of the endogenous stem cell compartment.

## Supporting information

S1 File S1_movie.avi. 2-D echo of patient with HLHS showing main chamber RV dysfunction and a small but contractile LV.
(AVI)
