# Correction: Cardiac regenerative capacity is age- and disease-dependent in childhood heart disease

**DOI:** 10.1371/journal.pone.0245808

**Published:** 2021-01-20

**Authors:** Alexandra Traister, Rachana Patel, Anita Huang, Sarvatit Patel, Julia Plakhotnik, Jae Eun Lee, Marel Gonzalez Medina, Chris Welsh, Prutha Ruparel, Libo Zhang, Mark Friedberg, Jason Maynes, John Coles

The seventh author's name is spelled incorrectly. The correct name is: Marel Gonzalez Medina.

## References

[1] Traister A, Patel R, Huang A, Patel S, Plakhotnik J, Lee JE, et al (2018) Cardiac regenerative capacity is age- and disease-dependent in childhood heart disease. PLoS ONE 13(7): e0200342 10.1371/journal.pone.0200342 30044800 PMC6059427

